# New Appliances and Things Medical

**Published:** 1899-07-29

**Authors:** 


### NEW APPLIANCES AND THINGS MEDICAL.

[We shall be glad to receive, at our Office, 28 & 29, Southampton Street, Strand, London, W.C., from the manufacturers, specimens of all new preparations and appliances which may be brought out from time to time.]

### KRONTHAL WATERS.

(Importers: Messrs. Schweppes, Limited, 53, Berners Street, W.)

These valuable mineral waters consist of three varieties, differentiated respectively by blue, green, and red labels. The blue label belongs to a pure table water of very agreeable qualities, very slightly mineralised, and highly sparkling. Being of extreme purity it is a safe and pleasant water, whether taken by itself or mixed with wines or spirits. The red label distinguishes a water very similar in quality, but more highly mineralised and of value in cases of gout and rheumatism where a pleasant muriated alkaline water is considered a desideratum. The water which bears the green label is sparkling and ferrugineous, for an iron water superior and more agreeable to the taste than most of well-known varieties. It will be found of value for cases of anæmia and debility, and should be taken regularly for some considerable period of time, as its action is mild and the percentage of iron correspondingly low.

### KUTNOW'S CARLSBAD POWDER.

(S. Kutnow and Co., Limited, 41, Farringdon Road, London, E.C.)

This preparation, which possesses for its full title the name of "Kutnow's Improved Effervescent Carlsbad Powder," while composed of the salts which have made the springs of Carlsbad famous, has been so ingeniously manipulated by laboratory processes that, though possessing all the therapeutic properties of the mineral water from the spring, it is rendered palatable and agreeably effervescent when mixed with water. For many cases of gout, rheumatism, indigestion, and allied conditions, Kutnow's powder, when taken regularly in moderate doses the first thing in the morning, has proved itself of real and lasting value.

